# CML: Evolution and design

**DOI:** 10.1186/1758-2946-3-44

**Published:** 2011-10-14

**Authors:** Peter Murray-Rust, Henry S Rzepa

**Affiliations:** 1 Unilever Centre for Molecular Science Informatics, Department of Chemistry, Lensfield Road, Cambridge CB2 1EW, UK; 2 Department of Chemistry, Imperial College of Science Technology and Medicine, London SW7 2AY, UK

## Abstract

A retrospective view of the design and evolution of Chemical Markup Language (CML) is presented by its original authors.

## The genesis of CML

**Figure 1:**
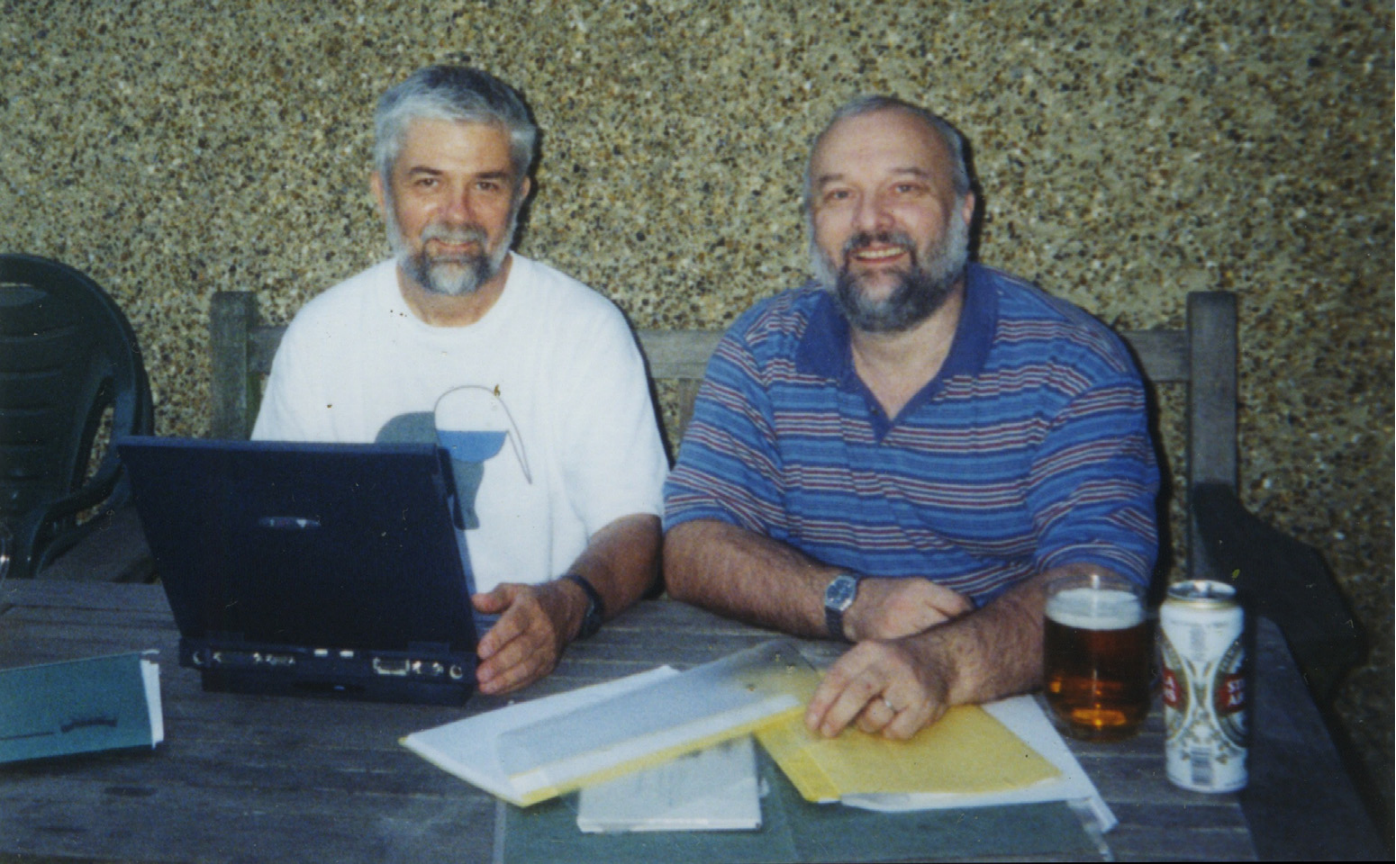
**The PMRz symbiote in a familiar environment**.

The modern online era has brought with it the need to rethink many of the mechanisms by which chemistry as a subject is researched, conducted and disseminated. This retrospective review describes how one infrastructure for doing so, CML or Chemical Markup Language, had its origins, and how the design evolved over a period of around 16 years (see also the accompanying spreadsheet for a breakdown of the historical timeline [Additional file 1]). In order to appreciate those origins, we start by describing the backgrounds and motivations of the present authors (Endnote 1) (Figure 1).

**Figure 2:**
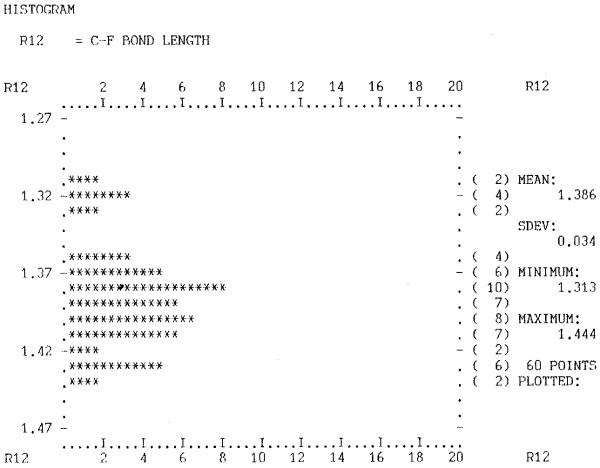
**Analysis of bond lengths (horizontal axis = frequency) from the Cambridge Crystallographic Database *ca*. 1995**. This has evolved into the bond length analysis tool in CrystalEye [60] which allows interactive clicking of points to bring up structures. (Note: Image is a scan of the original line printer output).

PMR recounts the early background to CML from his point of view: "What are the origins of CML? I think I go back to *ca*. 1980 when I was writing code to extend Sam Motherwell's great FORTRAN toolkit for the Cambridge database [1]- BIBSER (bibliographic search), CONNSER - the first and greatest chemical substructure algorithm, and GEOM78 - a geometry calculation tool. Between 1977 and 1980 I used to visit Cambridge (from Stirling) and work with Sam on extracting structures from the database and analysing them (Figure 2). There was a rough division of labour and ideas between us. I came with a number of ideas and Sam would modify CONNSER and GEOM to support these - literally within a day or so.

I took the problems back to Stirling and "integrated" Sam's output with SPSS [2]. I did the analysis on the floor of our living room with an acoustic modem [3] where the handset was plugged into rubber cups. It used to run at 110 baud. The sums were originally done at Cambridge (on Phoenix) but I ported the software to UMRCC (the Regional Computing Centre at Manchester) on a CDC 7600. The results were printed out on folding line printer paper on a boustrophedonic ASR33 teletype. I would then extract data by hand and enter them into the statistical programs, but gradually moved to doing the statistics remotely. Remote graphics was always difficult - we could get printer plots posted from Aberdeen but it took a week. So I generally evolved ASCII (line printer output) plots. One consequence is that during these sessions I had a lot of time to think about how to do it better. It was obvious the software had to be modular and I gradually got to thinking about modular data.

In 1981/2 I spent a sabbatical with Jenny Glusker [4] in Philadelphia and there developed a VAX-VMS version of the software. I extended this to plot aggregations of data in two and three dimensions. Again the idea of modular components was clear. I returned to start up molecular modelling/computer graphics in Glaxo and found myself working with a completely different set of data files -ChemX, MDL molfile, *etc*. I couldn't use these with my analysis code. This burgeoning of portable chemical computational systems had began in the early 1980s with a number of software products, mainly codes but also datafiles, being developed for the molecular graphics and computational chemistry community. In general, each resource developed its own representation of information, often referred to as a 'file format' or 'file type'. For example, by the mid 1980s there were probably fifty different file formats in chemistry including PDB, CSSR, MDL molfile and more specific program outputs [5] (Endnote 2). It was a major problem to convert between these formats, but despite some initiatives many software producers regarded the formats as proprietary and were resistant to ideas of inter-operability.

It seemed completely wasteful not to have a common format, so I started an activity within the Molecular Graphics Society [6] to systematize file types. In effect this was an attempt to build a chemical ontology. I didn't get much take up and there was active resistance from some software companies who regarded their formats as a commercial weapon.

During this period I had gradually advanced my language skills from FORTRAN to BBC-BASIC and C (part of this was through teaching the MSc in Birkbeck). So when C++ came along (late 1980s) I translated my approach to C++ and started to develop a toolkit/library. That's effectively when CML as a data modelling approach started."

HSR recalls his own early experiences in writing modular code: "In 1977, I had returned from the USA to Imperial College to continue my researches in quantum mechanical molecular modelling. Whilst at Austin in Michael Dewar's group, where I was learning about this area, I had encountered the famous ORTEP [7] program for displaying images derived from molecular coordinates, overkill of course for what I needed. So in 1977, armed with a Tektronix 4014 vector graphics terminal [8], I started to write a simplified molecular renderer (STEK) optimized for computational chemistry using FORTRAN code. A number of modules were aggregated, including a much simplified ORTEP-style molecular renderer with an appropriately semi-interactive interface for rotating the molecule into an effective projection, simple XY plotting routines, 2D contour and isometric plotting routines for potential energy surfaces and molecular orbitals, and various labelling and annotating routines. Data was read in from separate files (mostly the output of the MOPAC molecular orbital program) and written out into a (human- readable) single history file which could be used to restore the composite diagram. To separate the various data types, I developed a simple, rather *ad hoc *markup language, with a linear parser which could read back the data objects and associated display attributes to reconstruct the content model for the diagram. The project ground to a halt after about 10 years, largely because I had come to rely on a system graphics library (SIMPLE) targeting solely the Tektronix devices and the CDC computers my institute then operated. Remove this library and the hardware, and my (FORTRAN) program became very difficult to port (especially to the raster devices which were starting to replace the vector displays in the mid 1980s). It did teach me the value of markup (I was already used to word processing using troff [9]), of separating data elements into modular components, and in particular of stateless "round tripping", the ability to generate the output of a session in either a human- or a machine-readable manner that could be read back without loss, and in a reasonably error tolerant fashion, or with some components re-used for a different context. These absorbed concepts re-emerged some 10 years later when the ideas for CML started circulating.

I think my STEK program lasted perhaps 12 years, since I found that even in 1989, I was still using it [10,11] (good examples can be found in Figure one and Figures three & four respectively of these publications, which are not reproduced here for reasons of copyright). The molecule renderer had one feature unique to MO calculations not found in ORTEP, *i.e*. the ability to display the vibrational displacement vectors for a transition state, which was essential for understanding potential energy surfaces and the stationary points located.

The history of molecular orbital rendering is in itself an interesting one, since it introduces the connection between data, and its most effective representation. Hückel [12,13] was the first to apply MOs to "interesting molecules" such as benzene, but he famously never showed any diagrams. Dewar, starting in the early 1950s, did much to promote the use of molecular orbitals as a conceptual tool in chemistry, but he rarely provided quantitative representations in his articles. The great era of PMO theory in the 1950s was described using largely equations and tabulations of numbers rather than images [14]. Whilst I was a post doc with Dewar from 1974-1977, the group never in my recollection included MO wavefunctions derived using a graphical computer program in its publications! There was no idea to enhance the group's papers with such from Dewar himself, or the spark from anyone in the group to go find such a program (from *e.g*. QCPE [15] (if indeed any existed)). I introduced routine ORTEP plotting of molecular coordinates to the group's output, but never myself made the jump to rendering wavefunctions until inspired by an article on "orbital photography" in 1984 [16].

The relevance of MO rendering is that it highlighted in my mind the importance of data (calculated MO coefficients as numbers) and the need to interpret them semantically (which only visual rendering can do), in other words, the importance of both data and its appropriate rendering. Dewar's group could do the former, but never developed the expertise for the latter. Interestingly, Hoffmann's group did *both*, and acquired a major reputation in the field, including of course the Nobel prize for interpretations of pericyclic reactions [17]. Dewar always lamented his missing out on the Nobel prize, and arguably it was his inefficent application of data that might have been to blame.

One might argue that the reason the computational and the synthetic chemistry communities rarely mixed at that time is that the learning curve to understanding the tables of numbers that were being produced in theoretical articles was too steep for synthetic chemists. Perhaps images were also needed for impact? In 1984, the Rubinstein brothers developed ChemDraw (a 2D representational program), followed in 1986 by Chem3D, to be used at that time only on an overtly graphical computer (the Macintosh). This was one of a bevy of commercial programs of that era that started to address both the organic chemistry and modelling communities."

Back to PMR: "However the crystallographers had a much more unified view of the world. I continue to congratulate the International Union of Crystallography (IUCr) for its efforts in this area. In the mid 1980s the crystallographic community started to formalise its approach to small molecule X-ray diffraction. There was an active group, led by David Brown [18] aiming to create a self-defining format for crystallography- *Standard Crystallographic File Structure*[19,20]. It was essentially a data dictionary where a controlled vocabulary was created and specific semantics were added to items (*e.g*. data type). This approach was then taken further by the birth of the CIF initiative in 1990 and CIF is now the standard method of exchanging crystallographic information [21]. This was based on data supported by data dictionaries which themselves were constrained to a dictionary definition specification. I started to use the CIF approach to model my scientific world - this was long before XML but it was essentially isomorphic to XML - and it inspired much of the vision of CML.

I started with the most obvious components - geometry and numbers. These are still an integral part of CML (the "euclid" library). This was then extended to molecules, atoms and bonds and by *ca*. 1993 I had a set of objects. But I needed a way to display and manipulate them.

**Figure 3:**
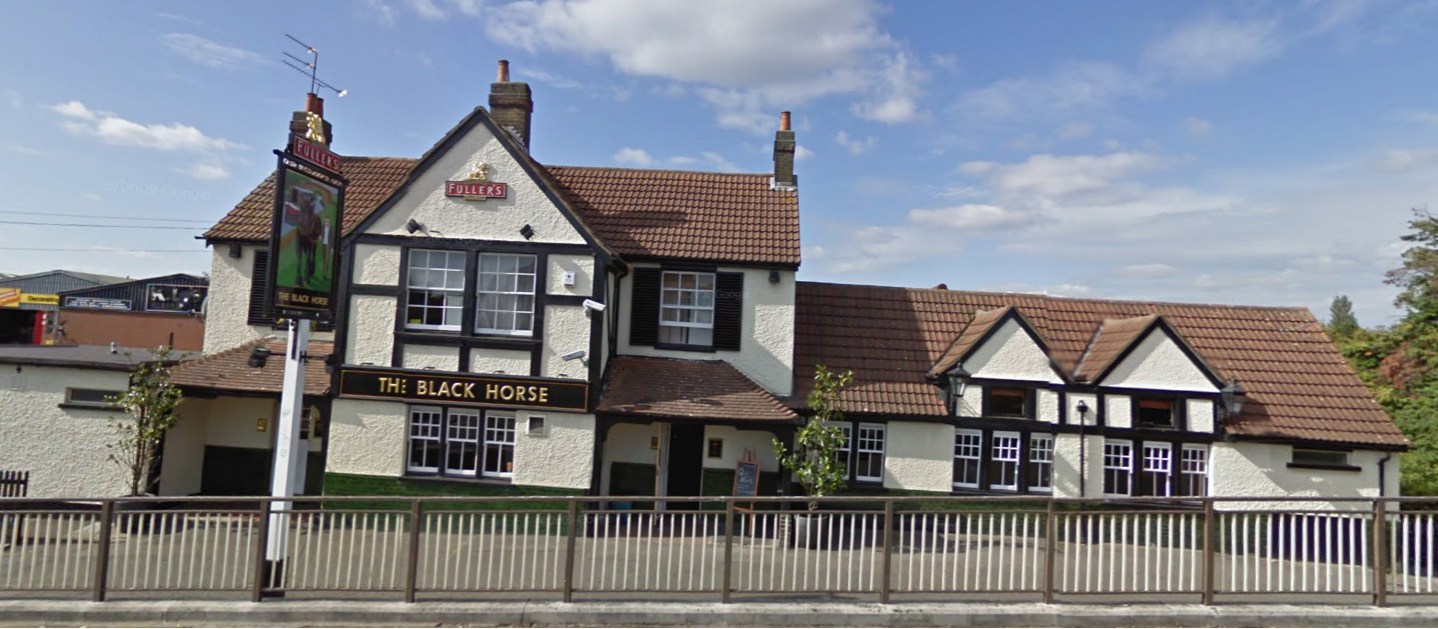
**The Black Horse pub at Greenford**.

At that stage I met Henry Rzepa. Henry remembers that probably around1993, the student chemical society at Imperial College invited me to give a talk. I chose the topic of crystallography, but in characteristic fashion, delivered a scintillating talk (Henry's description!) covering, well, probably almost all of chemistry! Henry chatted to me after my talk, and one of us must have mentioned the Internet. The topic might have been gophers (anyone remember them?) and what their potential was. Henry also visited me at Glaxo in Greenford around that time and we found we had a common interest in the Internet and its power for disseminating chemistry. It must have been about the time of the NCSA Mosaic browser [22] - 1993. This, in both our memories, is now immortalised by our working meeting in the Black Horse pub in Greenford in January 1994 (Figure 3). I had made initial explorations into a common format for chemistry, but it was the major adoption of HTML in 1993 and the announcement of the first World-Wide-Web conference (WWW1)[23] in 1994 that demonstrated that there was by then a critical mass of scientists and informaticians who wished to create semantic frameworks for information."

In the Black Horse, PMR and HSR agreed they would both attend the WWW1 meeting; HSR ran the session on chemistry and PMR one on biology. PMR: "We had an early version of RasMol [24] which ran on UNIX and Henry had prepared a demo, parts of which were eventually published [25]. We had it running the day before on a Silicon Graphics machine, but when we came back the next day someone had wiped the shared libraries to save space. We got the thing running again 5 minutes after Henry's talk [26] started.

The theme of WWW1 was, of course, the use of HTML (and HTTP) to create distributed information. Initially the focus was on HTML (with some discussion of the then very new MathML proposal by Raggett [27]), but during breakout sessions (or BoFs, birds-of-a-feather, as they were then called)[28] there emerged a realisation that each discipline would need to create its own approach to information that would be published and consumed in much the same way as HTML. Because it was in CERN and all HTTP sites at that stage were academic, the emphasis was all on science. Was MathML the way to carry maths in HTML? And if so, how could you do the same for chemistry? We didn't know how."

**Figure 4:**
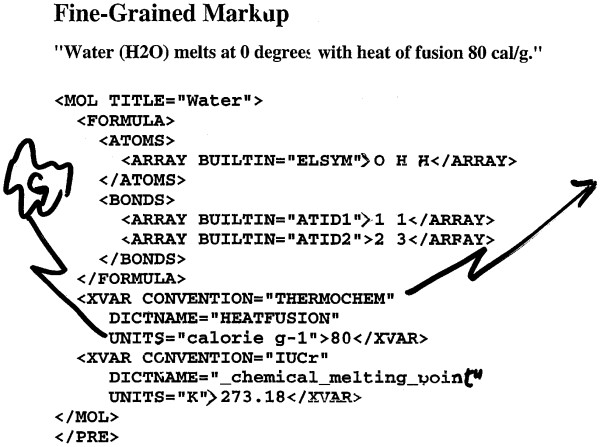
**Early CML markup, *ca*. 1995**. Many of the current concepts were prototyped at this level, such as the CONVENTION and DICTNAME (now dictRef). The BUILTIN attribute is now hard-coded as CML attributes. The XVAR notation has now become cml:scalar and all the elements and attribute values have become QNames. (Note: Image is a scan of an original overhead transparency with handwritten annotation).

The WWW2 conference (Chicago, October 1994) had a focus on the development of the HTML markup language and was noteworthy for the attendance of non-scientists and many commercial organisations, as well as a representation from chemists [29]. It also served to convince HSR that SGML [30] was the vehicle that would be adopted for non-textual information. As a result, PMR started implementing prototypes of chemical information using SGML and Tcl. At that stage SGML was complex and fragmented, to the extent that relatively few (if any) complete implementations existed. There were very few Open Source implementations and we were grateful to be able to use the nsglms parser [31] from James Clark which would take an SGML document and transform it into a structured representation (ESIS). At the same time, a COST project, with help from Joe English, allowed the ESIS to be processed in essentially the way that is now possible with the DOM [32], and PMR received much online help and guidance from Joe. By 1995, there was a prototype system where it was possible to read an early version of CML into a Tcl/Tk/COST processor and to display the structure of a molecule (Figure 4).

HSR recalls: "Checking through some ancient (*sic*) files on our web server, I discovered that we first went public with CML on 21 August 1995, in the form of an ACS poster [33]. I notice that it introduced the concept of what I had referred to earlier to as *data round tripping*. The idea was to formalise and normalise both the input and output of a computer program so that the latter could also reliably serve as an input. This was in fact implemented for the MOPAC program, and was a much more formal and structured expression of what I had earlier tried to do with the STEK graphical program. I was in Chicago, and Peter was back in the UK, at a terminal, waiting for comments from the audience on the poster to come flooding in! In fact, when we got to the hotel room that the ACS session occurred in, we discovered no trace of any Internet connection (anywhere) and could not communicate (Internet connections at conference venues only started becoming common from ~2006 onwards). Peter sat in an unrequited silence throughout the entire presentation! The poster was in fact presented as part of a session grandly entitled "Chemistry on the Infobahn".

**Figure 5:**
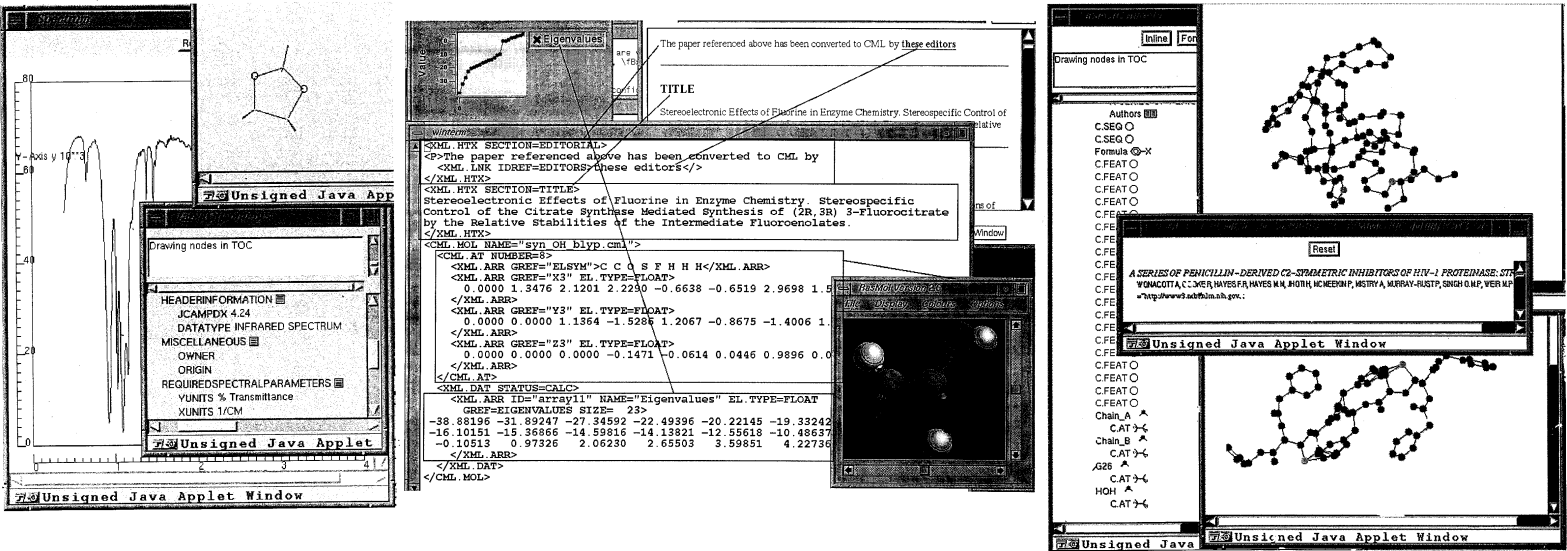
**JUMBO screenshots (*ca*. 1997) showing support for spectra, properties, molecular structure in 2D and 3D and a variety of applets and widgets**. Note the considerable change in syntax from the earlier picture; we have prototyped a namespace approach (*e.g*. XML.* and CML.*). These were later separated into CML and STMML [61]. The molecules and spectra had clickable locations so that peaks and molecules could be linked. (Note: Image is a scan of original overhead transparencies).

In 1995, HSR, PMR and Andrew Payne set up the Open Molecular Foundation (OMF) as a group to support and disseminate the creation of semantic chemistry using CML (SGML) as the infrastructure. This still required the combined operation of SGML processor executable and a wrapper which was being converted to Java 1.02. In late 1996, we became aware of the W3C XML project [34] (SGML on the Web) and joined the early discussion and working groups, one of which was located in central London (the Rembrandt Hotel meeting), which both PMR and HSR attended. There, Jon Bosak [35] declared that Java and XML were the foundations of the web! It became clear at this stage that CML should be based on XML, not SGML, and PMR was now working to an XML/Java representation in 1997 (Figure 5). A forum for XML developers was set up, which went live in February 1997 with a welcome post by PMR, and where many of the important developments involving XML were first discussed during the period of the operation of the list at its initial home (February 1997-December 1998) [36].

## The philosophy of CML

**Figure 6:**
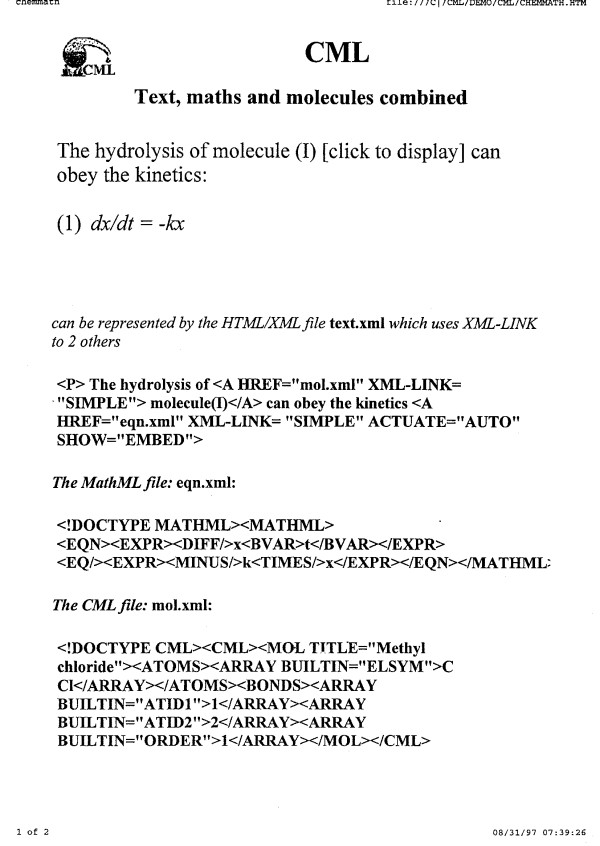
**A multi-namespace design from 1997 - the first use of the CML alembic logo**. This was before the XML Working Group created the current namespace syntax and while CML was still based on DTDs. (Note: Image is a scan of an original overhead transparency).

The primary purpose of CML has been and is to allow humans and machines to communicate chemical concepts without loss of semantic information. For example, a major role is to allow the output of one program to be converted into CML and input to another without loss. CML is also designed to create datuments, a combination of semantic text and non-textual information [37]. Our vision is that scientific publications should be represented semantically such that both humans and machines can consume them, again without loss. When CML is universally adopted for both these processes, then the large parts of the current discourse and information interchange in chemistry will be semantic. There is no reason why CML, in combination with other languages such as HTML, MathML, SVG, GML *etc*. (Figure 6) cannot then be used for at least the following:

• Ingestion of data into data- and knowledge-bases

• Extraction of data from knowledge-bases

• Journal articles

• Theses

• Suppliers catalogues

• Textbooks

• Regulatory documents such as patents and new drug applications

• Input to programs

• Output from programs.

The only technology capable of managing this at present (and probably for some time to come) is XML. It is widely used in publishing and CML can therefore be technically adopted for any of the document-like examples above. XML is also a primary method of marshalling input to databases and there are many tools which allow the construction of db schemas from XML schemas. In addition, however, CML has also shown itself to be valuable in the following areas:

**• A semantic infrastructure for physical science**. This is because none of the other scientific disciplines have developed markup support for dictionaries and units and so the CML constructs can support other areas.

**• A data structure for computation**. Although not originally intended for this purpose, XML is a very powerful data structure for internal data in computer programs (with some possible sacrifice in performance and memory size). Many of our programs use CML as their complete data representation and operate by adding to, removing from or modifying information on the DOM or infoset. For many applications this is a cleaner approach to passing data as everything is contained within one structure and there is no need for alternative storage of the same information. This, for example, is how all information in Chem4Word [38] and JUMBO [39] is held.

**• A computable object in its own right**. Because it is extensible and because computational semantics can be added to some of the elements, it represents a simple functional programming language. This is most developed in PolymerML [40] where a polymer can contain instructions for its own elaboration and the computation is carried out by repeatedly applying polymer extension semantics to the PML representation of the structure.

In designing CML, we have attempted to abstract the current common implicit and explicit concepts in mainstream chemistry. This is done by intensive and repeated analysis of chemical corpus linguistics. A common procedure is to take a recent journal article and to see to what extent CML can support the chemical concepts in that article. Similarly, we take the input and output of chemical programs and abstract new concepts and dictionary entries from those. In this way, we believe that CML is accessible to the chemical world and other scientists who use chemistry without a change in their concept structure.

## The evolution of CML

During the evolution of CML, we have been guided by the following factors:

**• The evolution of W3C recommendations and web technology and practice**. For example, when W3C introduced the XSD schema [41] recommendation we translated the CML DTD into XSD. When W3C introduced the XSLT [42] specification, we created a library of routines to process most of the CML elements. In similar ways, CML has reacted to incorporate SVG, RDF and OWL. These technologies themselves have had variable amounts of uptake. For example, until recently the main application of SVG was in mobile devices only, but now, 10 years after its launch, it is becoming mainstream in most browsers. We created an early Javascript tool (JUMBO-JS) which ran in the two current browsers in 2000 but which because of the rapid and uncontrolled changes in browser functionality no longer works and has been abandoned. However, it appears that JS is now reborn in a stable implementation and it is possible that we shall shortly create a CML reference. Similarly it has taken at least a decade for the concepts of RDF to mature and for a satisfactory toolset to start to appear. In these cases, CML had had to wait until there are clearly established and widely-used technologies that it can rely upon.

**• The interest of chemistry and the wider scientific community in markup languages**. Chemistry is recognised to be one of the more conservative scientific disciplines [43] and a new technology generally requires wide acceptance in other communities first or a powerful and determined commercial implementation. Although our work is well-known in the chemical informatics community, it is often said that "there is no demand for CML from our customers" with the result that a vicious circle ensues. Indeed some of the impetus comes from other subjects such as bioscience.

**• The Open Source (OS) community**. The OS community in chemistry is a relatively small part of the volume of software creation but it is highly visible and has a wide variety of offerings. Almost all OS chemical systems can read and/or write CML and there is a general agreement that these systems should converge to inter-operability. With the increasing rise of OS in general, and specifically in chemistry, this will be an important incentive to the adoption of CML.

**• Specific market applications**. The publishing industry is universally based on SGML and/or XML and so it is technically straightforward to incorporate CML in publications. The movement away from non-semantic output (such as PDF) is still slow but we believe that this is inevitable and again this will create considerable incentive to use CML. The small proportion of Open Access (OA) in chemistry means that it is very uncommon for scientists to extract information from the literature using machines and indeed many publishers expressly forbid this. As OA increases, we expect that the value of semantic information extracted from the literature will be seen to provide a large amount of additional value.

**• Toolset**. The chemistry community is likely to require a range of well-proven tools before it will adopt a new information technology. This takes time and/or financial investment before there is a perceived demand, but we believe that we are close to a situation where the value of this is starting to become apparent.

**• Regulatory and archiving**. There are several practices where XML is the recommended approach. In archiving material, XML can represent the semantics of a document and is frequently used by electronic archivists. In regulatory there is a requirement for many regulators to know the precise details of information in the doc and to be able to extract it rapidly. Therefore again XML is frequently required by regulators. Both of these pressures should lead towards a greater acceptance of CML.

## JUMBO

It has always been important that CML can be implemented in a reproducible and validatable manner. We are extremely reluctant to allow new elements or attributes in CML unless it can be demonstrated that they can be implemented and deployed in a large number of use cases without problems. It is surprising how often apparently small changes can severely disrupt the greater system. For example, allowing the explicitly declaration delimiter = ' ' as the delimiter for arrays and matrix elements, although apparently simple, has proved to be unworkable because of XML's normalisation of whitespace. We have therefore has to redesign the way in which the whitespace delimiter is used. Similarly in several cases we have used alternative values (such as 1 or S for single bonds) and this has resulted in a great deal of work and confusion. We use a deprecation mechanism to indicate that the reference implementations may cease to support outdated syntax. For this reason we have felt it necessary to build a reference implementation (JUMBO) which supports as far as possible all of the current elements and attributes and their semantics. Although JUMBO has now become a production system in much of our software, its primary *raison d'etre *is to show that information can be reliably processed, including round-tripping, and to provide reference examples of how various constructs should be used. JUMBO deliberately does not add large numbers of chemical methods (*e.g*. substructure search) but consumes these from other OS implementations.

JUMBO has been through six iterations, each more or less re-written from scratch. JUMBO1 used the rather primitive features in AWT1.0 to provide a hierarchical semantic browser of chemical documents (the name stands for Java Universal Molecular Browser for Objects). This was technically successful but not widely deployed because of the relatively small number of CML documents available at the time and the newness of Java. JUMBO2 was a development-only version and mapped the CML elements onto editable widgets such as textboxes, lists and molecules. It used an early version of Swing and because of the difficulties in that was never formally distributed.

JUMBO3 returned to the browser concept and displayed a CML document in a series of windows (rather similar to the current Bioclipse [44] tool). There was a brief flirtation with Java 3D for molecular display but we reverted to including Jmol [45] as a callable window where required. At this stage it became clear that the continued development of the schema made it very difficult to keep the specification and the software in sync. In JUMBO4 we attempted to use the W3C DOM implementation as our data structure. This turned out to be very problematic as the library was not designed for subclassing and all elements had to delegate to the W3C DOM object. There were several other problems with the W3C DOM including the lack of any XPath [46] functionality and towards the end we moved to the much more satisfactory XOM from Elliotte Rusty Harold [47]. This has proved to be an extremely useful and reliable choice as it not only provided a relatively simple view on the DOM but it manages concepts such as namespaces extremely well.

As more elements were added to the schema, maintenance became a real problem, with 100 elements and 100 attributes admissible in various combinations. Content models which allowed and constrained combinations of child elements became very complex and unmanageable, and it was almost impossible to write consistent code. Therefore, in JUMBO5 we resorted to auto-generation of the basic code from the schema. This meant that when new elements were added the code was regenerated from the incremented schema.

JUMBO6 was primarily a refactoring of the functionality of JUMBO5 resulting in a clean design for the implementation of converter functionality. We have continued to modularise so that now there is a basic CML XOM (where most methods simply represent accessors and mutators). JUMBO6 is a set of tools providing additional chemical functionality, especially those for manipulating the DOM, and a separate large library of JUMBO-Converters which extract the output from programs and documents and convert it into hierarchical CML docs. At this stage, we also developed Chem4Word, which uses a fully validated convention of CML ("CMLLite"). This took a much more lightweight approach to content models and created the concept of validation. In the Chem4Word system all potential input is validated against a rich combination of XSLT expressions which are far more powerful than XML schema (XSD). This is now a continuing philosophy.

## Code-driven CML Design

As part of the CML philosophy we have strongly adopted the 'rough consensus and running code' stock (originally coined by David Clark in 1992)[48]. An excellent example of the value of developing code in parallel with a specification was given in the early days of XML. The initial design included a nearly-full implementation of SGML parameter entities. At this time two or three prototype XML parsers were being developed (Norbert Mikula, Tim Bray, PMR) and it became clear that the implementation of the full parameter entity model was a major effort for relatively little reward, and it was therefore dropped from the specification. We have found the same in CML, sometimes only surfacing several years after a feature was introduced. The abstraction of data into scalars, arrays and matrices with associated data types (XSD data types) has been a considerable effort but its successful deployment for several years has shown that the design is both implementable and valuable.

Similarly the design of the dictRef attribute value has evolved to support qualified names (QNames). This was not originally driven by W3C architecture but by the need to uniquify entries in dictionaries but it became clear that dictRefs and other pointers/links were isomorphous with the URI concept (which only became relatively widely-deployed about 5 years ago). The simultaneous development of code and specifications meant that we could implement early versions of QNames/URI and these are now a major feature of CML.

It is sometimes impossible to tell what the effect of a schema design will be before deployment. A particularly difficult problem has been white space. In many cases (such as in formatted files like PDB), whitespace is extremely significant ('CA' is calcium whereas 'CA' is a C-alpha). XML attributes will normalise whitespace (trimming strings and replacing all internal whitespace by single space characters). The use of attributes and #PCDATA content have different consequences in XML processors, which cannot easily be predicted before widespread deployment.

Another major feature in the design was the need to validate combinations of elements and attributes. At one stage, PMR was approached by a group of pharma and related companies, to create a specification of CML for the Object Management Group (OMG)[49]. Hand-coding the combinations of attributes and elements proved impossible (this is effectively a sparse 100*x*100 matrix) and it was clear that automated methods of validation and code-generation could be necessary. JUMBO4.6 therefore generated code from the schema model rather than requiring it to be hand-coded. However, the W3C DOM technology was not well-suited to this, leading to the adoption of the simpler XOM model. None of this had an immediate effect on the surface schema of CML but has had deep influences on the subsequent design.

**Figure 7:**
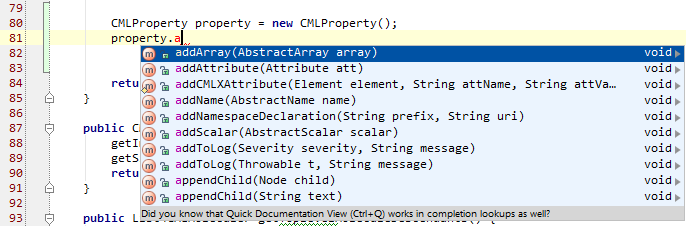
**The auto-complete functionality in IDEs is underpinned by the content model approach**.

As a result of this, we developed the attributes and elements largely independently; in other words, attributes are generally not context-specific. The commonest attributes are id, title, dictRef, and these can be found on essentially every element. Content models are now used with optional components to generate convenience methods for managing child elements. An obvious benefit is in providing auto-completion in IDEs (*e.g*. the CMLProperty class might prompt for 'addArray') (Figure 7).

However, with increasing deployment to different areas of chemistry, it became clear that it was going to be impossible to find universal content models for most elements. This is due to not only the diversity of chemistry but also the different ways that chemists might wish to organise information. A molecule might have one or more spectra as children (representing that these are associated analytical data). Alternatively a spectrum could have one or more molecules as children representing that these correspond to different peaks. It was this type of experience that led us to propose an extremely flexible content model, constrained by the use of convention rather than XSD technology.

The context-free attribute design has been largely successful. In a few cases, common words such as 'type' have been used polymorphically, and have incompatible enumerations. Thus the elements '**spectrum type **= "NMR"' and '**reaction type**= "reversible"' actually use different attribute types (spectrumType and reactionType), and it was for lexical convenience that both of these mapped onto the string 'type'. This approach has caused considerable implementation problems, and, were we to re-factor the schema, we would make these unique.

The use of enumerations (*e.g*. xsd:list) has been beneficial and problematic. In some cases the enumeration is fixed (*e.g*. periodic system of the elements); in others (*e.g*. spectrumType) there are a number of common values but we can anticipate new types as a result of scientific discoveries and new instrumentation. The current design tried to satisfy this by giving a list of common enumerated types but also adding the possibility of a user-defined type (using the XSD union approach). This has proved extremely complex to implement and has brought relatively little value. In future, we would support semi-controlled enumerations through conventions rather than through XSD technology.

There is a special case with XSD data types. If we adopted all (*ca*. 48) of these, then there would be a possible commitment that implementers had to implement all of them. If the XML is being used in an XSD system such as entry to a database this is manageable, but for the more flexible requirement of heterogeneous CML it becomes a major burden. Therefore we have arbitrarily selected a small number of data types (xsd:string, double, integer, date, boolean). None of these puts any restriction on how CML holds the information and a double can be of arbitrarily large precision.

In general, giving multiple options, even apparently simple choices, for values is an extreme burden on implementers. A bond order was originally allowed to have order = '1' or order = 'S'. Managing both of these simultaneously is a remarkably high burden and we have deprecated and almost eliminated the use of numeric bond orders.

The requirement that all physical quantities have dictRefs and units of measure has been extremely successful. The only area where this is poorly defined is for atomic co-ordinates in molecules. By default, these are in Ångstrom units and there was no provision for specifying other units. With the development of many CML systems in computational chemistry and physics we need to be able to support nm, pm and atomic units (Bohr). There is currently no very clean way of doing this and it requires the units attribute to be added to molecule which is semantically illogical but currently just about manageable.

It is also difficult to know at the start how many container elements should be used. For common elements such as property, parameter and molecule there are specific container elements (*List, *e.g*. moleculeList). For others, we rely either on the implicit ordering that XML supports or provide a generic cml:list element. The latter has proved to be extremely valuable in interpreting the data in computational chemistry.

The increasing availability of XPath-based technology has had a major positive effect on the possible flexibility of the organisation of elements and attributes. For example, in documents as large as several megabytes, it is possible to use XPath expressions to locate, delete, change, add and move (sort) components. A typical computational chemistry logfile or a crystallographic experiment (CIF) in CML can be manipulated with great power and flexibility. This means that content models are almost irrelevant whilst XPath-assisted conventions are a major tool in normalising and re-purposing chemical information.

## Validation

**Figure 8:**
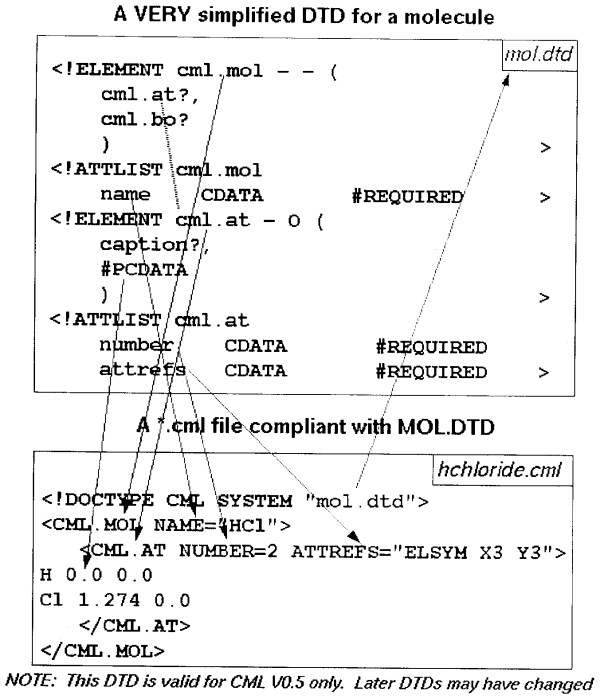
**A version of the CML DTD (in SGML) from *ca*. 1996**. Note the early development of a namespace philosophy although there was no technology to support it at the time. (Note: Image is a scan of an original overhead transparency with handwritten annotation).

The original purpose of SGML was to act as a machine-enforceable contract between an author and a typesetter. SGML tools could indicate that a document was valid or invalid and each party would know whether it was their responsibility or that of the other. The DTD therefore also acted as a specification against which compliant software could be written (Figure 8). This idea is very much at the heart of CML and represents the first major infrastructure for validating chemistry (crystallography excepted).

Many of the problems of software and data in chemistry can be traced to the lack of a validation system. Without validation, the author of a program cannot easily write conformant software if the input is variable; similarly the author cannot know whether an input is fit for purpose. Unfortunately the past 30 years have seen a wide variety of formats each with a wide variation in conformance. For example, there is no accepted 'standard' for PDB files and many program authors have modified this format for purposes other than managing protein crystal structures (*e.g*. computational chemistry output). As a result, many programs corrupt information because they cannot validate the input, and they make unwarranted assumptions. By ensuring that input and output are both valid or validatable, it becomes possible to link processes and ensure no semantic corruption or loss.

There are many implicit assumptions about the representation of chemistry that cause semantic problems. For example, very few datafiles state the units of measure of scientific quantities and there are frequent assumptions about the existence of hydrogen atoms. There is much confusion between 2D and 3D coordinates and few systems can hold both at the same time. A major purpose of CML is to make sure that all chemical information is validatable and that the rules for this validation are openly visible. Most recently we have constructed Chem4Word and the CMLLite specification [50] which shows that complete validation of input and output chemistry is possible even in complex systems. In that case, the validation is carried out by stylesheets/XPath which has most of the power that is required.

## Community-driven CML Design

CML has always been a community project in that its progress has been visible and it has been possible for anyone to provide feedback. It is not however a community-managed project and is best described by the BDFL model [51] ('Benevolent Dictator For Life'; *cf*. Linux). We feel this is necessary to ensure a consistent vision for the infrastructure and we have also felt that until this achieved stability it was unreasonable to expect others to volunteer contributions when they might be discarded at any stage. There have now been several sub-projects in CML where the community has been actively involved in design, including "CMLReact", "CMLSpect" and some aspects of "CMLComp" ("compchem"). There have been very few major additions to CML in the last three years despite its increasing deployment and we therefore feel that the central architecture is fit for purpose and can be extended by the community in a variety of ways that suit their own needs (mainly through dictionaries and conventions).

## Foreseeable evolution of CML

Our explorations in a wide range of chemical documents and documents containing chemistry have shown that CML is capable of managing disciplines as far-ranging as atmospherics, minerals, enzymes, analytical and computational chemistry. The immediate vision is that the world would benefit enormously from having this material available in CML. The barriers are almost all cultural; few chemists see the merit of this and therefore few if any publishers of chemistry make any provision for doing this. We believe that the increasing value of semantic material (*e.g*. Linked Open Data, LOD) will gradually show the community that this is enormously important.

There are four main methods of creating semantic chemistry: a) human authoring (as in conventional articles, reports, laboratory notebooks etc.), b) conversion of chemical data from legacy formats, c) creation of semantic chemistry through computer program output and d) machine extraction of chemistry from unstructured and semi-structured material (*e.g*. electronic chemistry publications). We see the following opportunities and barriers to each of these, listed below. There is a "chicken and egg" aspect to this. We are frequently told that there is "no demand for CML" and as a result people do not create tools that read or produce it. In several cases we have attempted to overcome this by creating believable prototypes in these areas.

**a) Human authoring **is likely to happen when there is a sufficient range of semantic editors. We have created Chem4Word to show that this is possible but it needs an acceptance by the community that semantic authoring is something that is desirable in an editor. Although the default authoring tools are currently Word and LaTeX, we expect that web-based tools such as GoogleDocs and EtherPad will lead to much more attractive environments in which scientists will create documents. Two good examples of this are Southampton's Blog3 software [52], where a blogging platform is used to create chemical documents, and Peter Sefton's Scholarly HTML initiative [53], showing that modern scholarship, including science, should be managed through HTML and not doc/pdf.

**b) File format conversion**. The problem of converting between different file formats has been largely solved by the Blue Obelisk community [54]. Our own JUMBO-Converters will convert many of the common formats (mol, smi, pdb, cif, cdx *etc*.), especially the more complex ones, into structured CML without semantic loss. There is no technical barrier to rapid and widespread uptake by the chemical community.

**c) Conversion of legacy to include semantic content**. Many programs such as quantum chemistry and molecular dynamics produce logfiles originally aimed at printing on fan-folded line printer paper. We have shown that it is possible to intercept all output statements and convert them to CML (*e.g*. for SIESTA, CASTEP, MOPAC, DL_POLY). These outputs have been used in the computational minerals and materials communities and we have created libraries (*e.g*. FoX [55]) to make this process easy. However, at present most codes have not adopted this and we have therefore written a series of converters based on a declarative parsing technology (JUMBO-Parser) which allows for very high (greater than 95%) precision and recall of the structure and semantics of the documents. These are being developed by the Quixote community [56] for computational chemistry programs and are generally adaptable to any program which produces combined text and numeric output.

**d) Machine-extraction of chemistry**. Our OSCAR program [57] has high success (80-90% precision and recall) in extracting chemical entities from unstructured text. The success rate depends on the specific domain but ranges from atmospheric chemistry through biomedical to synthetic chemistry. In all text-mining areas, it is much more difficult to extract processes, relationships and sentiment from documents. However, chemical syntheses are reported in such a formulaic manner that we can extract a very high degree of the underlying semantics of the chemical reactions. The primary barrier to this are the legal prohibitions demanded by mainstream chemical publishers which are generally agreed by subscribing institutions, meaning that it is a contractual violation to undertake text-mining activities. BMC journals are an exception (being published as CC-BY) but there is relatively little mainstream chemistry published in BMC at the moment. We have been able to show that we can extract chemistry from patents, although the quality of many of these is not perfect and there are errors due to transcription. Recently the British Library has argued strongly for the reform of intellectual property laws to allow text-mining for scientific and related purposes [58], and if this were to happen, there are enormous opportunities for CML technology to provide near-universal semantic chemistry.

Assuming that the cultural, political and legal barriers are removed, it is very cost-effective to produce all chemical information using CML. We have shown that, at near-zero cost, the whole of published crystallographic data can be converted into semantic form (250, 000 structures in CrystalEye). Similarly, we have read 100, 000 patents and extracted reactions; others have used OSCAR to add semantics to information from Medline. If the chemical community can agree identifier systems for the components (molecules, reactions, spectra etc.), this would create a huge resource of chemistry to be added to the LOD cloud. We would expect this to be indexed on compounds, substances, reactions, spectra, crystal structures and many aspects of physical chemistry. By creating dictionaries, ideally with the involvement of authorities such as IUPAC and IUCr, we then have a comprehensive semantic framework with a simple computable ontology. It is difficult to predict exactly what the benefits of this will be, but they will be massive. LOD provides for linking between disciplines, *e.g*. the concentration of chemicals in the atmosphere at different times and geographical locations. It allows for systematics within a sub-discipline (*e.g*. comparing all published synthetic procedures and analysing these for the potential value of reaction conditions, catalysts etc.) It allows experimental data (*e.g*. crystal structure) to be used to calibrate computational approaches such as quantum mechanics. With the addition of natural language, this becomes a human-accessible resource where we can ask simple powerful questions to the machine such as "find me all spectra which contain NMR shifts below zero and which do not contain metals" or "find me all solvents involved in reactions above their boiling point". In computational chemistry, CML can largely automate the process of creating multiple jobs through parameters sweeps and analysing and searching the outputs. Indeed, it is reasonable to see program manuals being replaced by CML dictionaries appropriate to that program, and understandable both by machines and humans.

## Sustainability

Every semantic project must address its sustainability. In the past CML has been highly dependent on its two authors. The technical resource to support it in its current form is now relatively modest, so that in principle it could be forked or continued (the "Dr. Who model of OS"[59]) were the current authors to become inactive. It has been implicitly and explicitly endorsed by a range of companies and organisations including the IUCr, Unilever Research, Microsoft Research and the National Cancer Institute of the US National Institutes for Health. There is a wide range of CML-compliant software and a large number of examples. Its technical sustainability therefore seems assured, and its political sustainability is beyond the scope of this article. We are confident that in the not too distant future publishers such as BMC will enthusiastically accept contributions consisting partly or mainly of CML.

## Competing interests

The authors declare that they have no competing interests.

## Authors' contributions

PMR and HSR both developed CML and wrote the manuscript.

## Endnotes

### Endnote 1

CML is a joint creation of the two authors over many years. Some of this paper is written in the first person, other sections refer to 'us' or 'we'. Anything that appears to refer to one or other in the singular should be mentally replaced by 'the PMRz symbiote'.

### Endnote 2

Open Babel represents an almost comprehensive identification of these formats, which in 2011 has reached 113 formats in chemistry.

## Supplementary Material

Additional file 1 **Timeline**. Spreadsheet of the Chemical Markup Language design evolution timeline. Click here for file
